# Trypsin- and low pH-mediated fusogenicity of avian metapneumovirus fusion proteins is determined by residues at positions 100, 101 and 294

**DOI:** 10.1038/srep15584

**Published:** 2015-10-26

**Authors:** Bingling Yun, Xiaolu Guan, Yongzhen Liu, Yanni Gao, Yongqiang Wang, Xiaole Qi, Hongyu Cui, Changjun Liu, Yanping Zhang, Li Gao, Kai Li, Honglei Gao, Yulong Gao, Xiaomei Wang

**Affiliations:** 1 Division of Avian Infectious Diseases, State Key Laboratory of Veterinary Biotechnology, Harbin Veterinary Research Institute, Chinese Academy of Agricultural Sciences, No. 427 Maduan Street, Nan Gang District, Harbin 150001, Heilongjiang Province, PR China

## Abstract

Avian metapneumovirus (aMPV) and human metapneumovirus (hMPV) are members of the genus *Metapneumovirus* in the subfamily *Pneumovirinae*. Metapneumovirus fusion (F) protein mediates the fusion of host cells with the virus membrane for infection. Trypsin- and/or low pH-induced membrane fusion is a strain-dependent phenomenon for hMPV. Here, we demonstrated that three subtypes of aMPV (aMPV/A, aMPV/B, and aMPV/C) F proteins promoted cell-cell fusion in the absence of trypsin. Indeed, in the presence of trypsin, only aMPV/C F protein fusogenicity was enhanced. Mutagenesis of the amino acids at position 100 and/or 101, located at a putative cleavage region in aMPV F proteins, revealed that the trypsin-mediated fusogenicity of aMPV F proteins is regulated by the residues at positions 100 and 101. Moreover, we demonstrated that aMPV/A and aMPV/B F proteins mediated cell-cell fusion independent of low pH, whereas the aMPV/C F protein did not. Mutagenesis of the residue at position 294 in the aMPV/A, aMPV/B, and aMPV/C F proteins showed that 294G played a critical role in F protein-mediated fusion under low pH conditions. These findings on aMPV F protein-induced cell-cell fusion provide new insights into the molecular mechanisms underlying membrane fusion and pathogenesis of aMPV.

Avian metapneumovirus (aMPV) belongs to the genus *Metapneumovirus* in the subfamily *Pneumovirinae* of the family *Paramyxoviridae*^1^. aMPV causes acute rhinotracheitis in turkeys of all ages and swollen head syndrome in chickens, resulting in a major economic burden for poultry farmers^2^. Since aMPV was first detected in South Africa in 1978, aMPV has been reported in many countries^3^. Phylogenetic analyses have categorized the aMPV strains into four subtypes, termed A, B, C, and D, which are sometimes designated as aMPV/A, aMPV/B, aMPV/C, and aMPV/D, respectively^4^. Metapneumoviruses have recently been subclassified into two types based on phylogenetic analysis: type I metapneumoviruses, including aMPV/A, B, and D, and type II metapneumoviruses, including aMPV/C and human metapneumovirus (hMPV)^5^.

The life cycle of enveloped viruses is initiated after binding to a specific receptor(s) on the host cell surface, followed by membrane fusion, which is mediated through one or more viral glycoproteins^6^,^7^,^8^. In general, the viral attachment proteins (G, H, or HN, depending on the virus) of paramyxoviruses, such as Newcastle disease virus (NDV), human parainfluenza virus type 3 (hPIV3), and human parainfluenza virus type 5 (hPIV5), cooperate with fusion (F) proteins during membrane fusogenic events^9^,^10^,^11^,^12^. aMPV, hMPV, and human respiratory syncytial virus (hRSV) have three glycoproteins, including F, small hydrophobic (SH) and G proteins; however, the SH and G proteins of aMPV, hMPV, and hRSV are not essential for viral replication or the activation of membrane fusion in cultured cells^13^,^14^,^15^,^16^,^17^. Moreover, the expression of aMPV, hMPV, and hRSV F proteins alone results in cell-cell fusion^18^,^19^,^20^,^21^,^22^, suggesting the fusion mechanism of these viruses might be unique among paramyxoviruses.

The F protein of aMPV and hMPV is a class I viral fusion protein^23^,^24^,^25^,^26^ and is synthesized as the biologically inactive precursor, F0, which must be cleaved into F1 and F2 to become fusion competent. Viral and cell membrane fusion facilitates virus entry into the target cell, and cell-cell fusion leads to syncytium formation^27^,^28^,^29^,^30^,^31^,^32^. Previous studies have established that trypsin is required for the *in vitro* propagation of hMPV strains^33^,^34^,^35^,^36^,^37^, indicating that trypsin might be critical for the cleavage of hMPV F proteins. Nevertheless, conflicting data have shown that some hMPV strains grow *in vitro*, independent of trypsin^19^. Schickli *et al.* demonstrated that the amino acid substitution S101P in the putative cleavage motif of hMPV F protein is a major determinant for trypsin-independent growth in Vero cells, and the S101P mutant can be cleaved in the absence of trypsin^19^. Wei Y *et al.* reported that trypsin is required for the induction of aMPV/B F protein-mediated cell-cell fusion, but this enzyme is not crucial for the fusion mediated through aMPV/A and aMPV/C F proteins^18^.

Viruses are generally classified into two entry categories: low pH/endocytic entry and neutral pH/plasma membrane entry^38^,^39^,^40^,^41^. Paramyxoviruses promote membrane fusion at the plasma membrane at neutral pH^20^,^38^,^42^. However, numerous reports have shown that the F proteins of some strains require stringently low pH for cell-cell fusion^38^,^39^,^43^. Schowalter RM *et al.* demonstrated that hMPV uses the low pH of the endocytic pathway; the low pH environment stimulates F protein, thereby triggering membrane fusion and syncytium formation^20^,^38^. Although some hMPV F proteins require low pH for efficient fusion, low pH does not typically activate hMPV F proteins for membrane fusion^39^. Hence, the dependency of hMPV F proteins on low pH for membrane fusion remains controversial. Both aMPV and hMPV belong to the genus *Metapneumovirus*, and the fusogenic mechanism of the F proteins of these viruses is unique from those of other paramyxoviruses^18^.

We used syncytium formation assay^44^,^45^, reporter gene assay^8^,^39^, and biotinylation and western blot analysis^38^,^46^ to characterize the fusogenicity of aMPV F proteins. The data demonstrated that the membrane fusion activities mediated through aMPV/A, aMPV/B, and aMPV/C F proteins are distinct when exposed to trypsin and/or low pH. In addition, the amino acid residues at positions 100, 101 and 294 were identified as major determinants of the trypsin- and pH-dependent or -independent phenotypes in the membrane fusion stimulated through aMPV F proteins.

## Results

### Effect of trypsin on the fusogenic activity of aMPV F proteins

**Figure 1:**
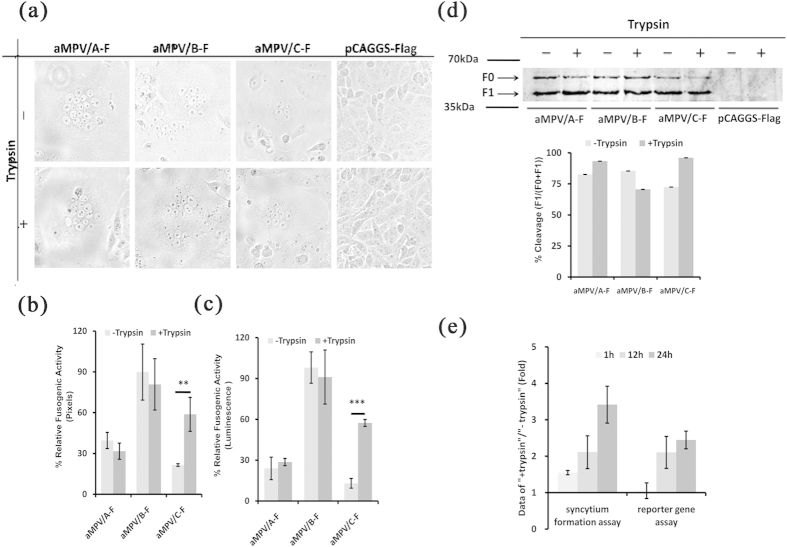
Effect of trypsin on membrane fusion induced through aMPV F proteins. (**a**) aMPV F proteins induced syncytium in Vero cells with or without trypsin treatment (magnification: 100×, 8 cm ×8 cm as shown). (**b,c**) The fusogenic activity of aMPV F proteins with or without trypsin treatment was analyzed based on syncytium formation and reporter gene assays. *P* < 0.05 and *P* < 0.005 are respectively presented as **and ***. (**d**) The cleavage of aMPV F proteins was analyzed using biotinylation and western blot analyses. (**e**) The effect of the duration of the trypsin treatment on the fusogenic activity of aMPV/C F protein. The data represent the ratio of treated vs. untreated aMPV/C F protein.

Syncytium formation induced in Vero cells by F proteins is typically used as an indicator of fusion to estimate the membrane fusogenic activity of F proteins^19^,^20^,^39^,^47^. The effect of trypsin on the fusogenicity of aMPV F protein was initially determined. Vero cells were transfected with aMPV/A-F, aMPV/B-F, or aMPV/C-F and subsequently treated or mock-treated with trypsin. At 48 h post-transfection, the results showed that syncytium formation was extensively observed in cells transfected with each of the three plasmids carrying the F proteins indicated above, irrespective of the presence of trypsin (Fig. 1a). In addition, no syncytium was formed in cells transfected with empty vector carrying Flag tag, regardless of treatment with trypsin (Fig. 1a). Moreover, the syncytium induced through aMPV/C F protein after trypsin treatment increased in number (data not shown) and size (Fig. 1a), but these differences were not observed in cells expressing aMPV/A and aMPV/B F proteins. We further investigated the impact of trypsin on the fusogenic activity of aMPV F proteins using a quantitative assay, and the addition of trypsin did not result in a notable difference in aMPV/A or aMPV/B F protein fusogenicity compared with the corresponding samples that did not receive trypsin treatment (Fig. 1b,c). However, the fusogenic activity mediated through aMPV/C F protein was remarkably increased in the presence of trypsin compared with that in samples without trypsin treatment (Fig. 1b,c). Overall, the results obtained from the two quantitative assays were consistent with those obtained from the syncytium formation assay described above. Based on these findings, we next determined whether aMPV F protein is cleaved under the equivalent conditions described above because previous studies have suggested that the cleavage of F protein is required to induce cell-cell fusion^9^,^39^,^48^,^49^. Thus, the expression of biotinylated aMPV F protein on the cell surface was determined through western blotting using an anti-Flag antibody, as previously described^38^,^46^. As shown in Fig. 1d, the F proteins of all three aMPV subtypes were efficiently cleaved in the absence of trypsin (Fig. 1d). The addition of trypsin did not facilitate the cleavage of aMPV/A and aMPV/B F proteins, whereas trypsin slightly enhanced the cleavage of aMPV/C F protein (Fig. 1d). Taken together, these results showed that (i) the F proteins of all aMPV subtypes tested induced cell-cell fusion in the absence of trypsin and (ii) unlike the F proteins of aMPV/A and aMPV/B, aMPV/C F protein enhanced cell-cell fusogenic activity in the presence of trypsin, likely reflecting the efficient cleavage of aMPV/C F protein.

The results also revealed that aMPV/B (VCO3/60616 strain, accession number: AB548428.1) F protein induced cell-cell fusion, independent of trypsin. This finding is inconsistent with a previous report showing that aMPV/B (Shaoxing strain, accession number: JN224985.1) F protein mediates cell-cell fusion in a trypsin-dependent manner^18^. Unlike the low fusogenic Shaoxing F protein^18^, VCO3/60616 F protein is hyper-fusogenic, inducing syncytium in the absence of trypsin (Fig. 1a). Furthermore, aMPV/B propagated in Vero cells, BHK-21 cells, and DF-1 cells in the absence of trypsin (data not shown).

Results showing that the fusogenic activity of aMPV/C (Colorado strain, accession number: AY590688.1) F protein was profoundly increased upon trypsin treatment (Fig. 1b,c) are not consistent with those of Y. Wei *et al.*, who illustrated that trypsin treatment did not enhance the cell-cell fusogenicity induced through F proteins of the same strain^18^. Based on previous studies^19^,^38^, trypsin treatment was conducted for more than 12 h, whereas Y. Wei *et al.* performed trypsin digestion for 1 h. We hypothesized that the discrepancy in the results reflects these differences in the duration of trypsin treatment. To test this hypothesis, we examined the fusogenic activity induced through aMPV/C F protein at different time points after trypsin treatment. As shown in Fig. 1e and consistent with Wei Y *et al.*^18^, the fusogenic activity of aMPV/C F protein exposed to trypsin for 1 h was equivalent (in the reporter gene assay) or indistinguishable (in the syncytium formation assay) to that without trypsin. In addition, consistent with previous reports^19^,^38^, aMPV/C F protein exposed to trypsin for 12 or 24 h exhibited significantly increased fusogenic activity compared with that without trypsin treatment (Fig. 1e). These results confirmed the hypothesis that this disparity reflected differences in the duration of trypsin treatment.

### The residues at positions 100 and 101 are determinants for aMPV F protein-mediated membrane fusion in the absence of trypsin

**Figure 2:**
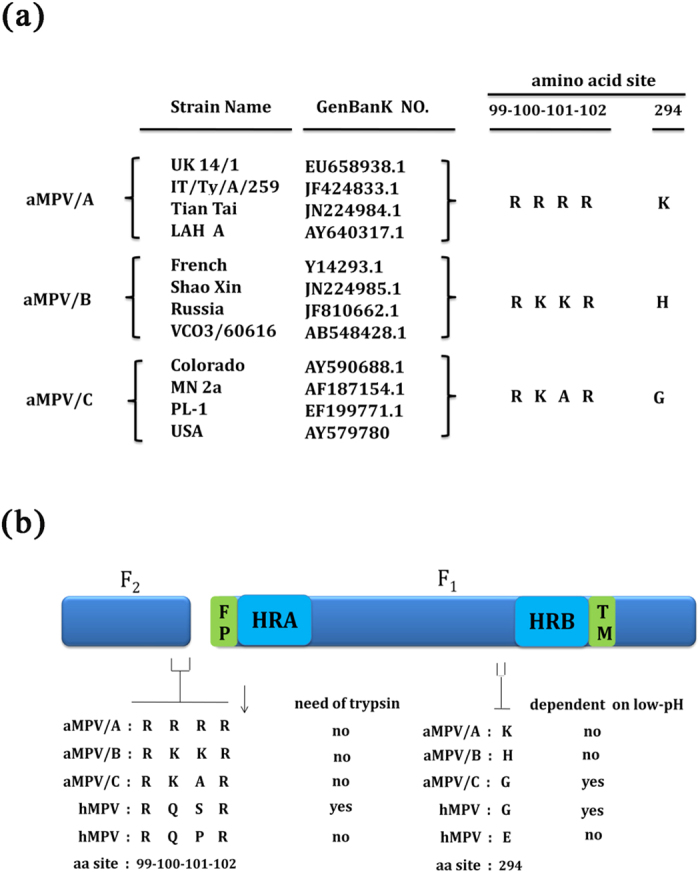
Comparison of the sequences and model structures of aMPV F proteins. (**a**) The amino acid positions affect the fusogenic activity of aMPV F proteins exposed to trypsin or low pH according to the alignment with sequences of hMPV F proteins (hMPV strain CAN97–83, NL/1/00, and NL/1/17)^20^,^38^,^39^. (**b**) A schematic of the aMPV F protein. The aMPV F protein is proteolytically cleaved into fragments F1 and F2.

**Figure 3:**
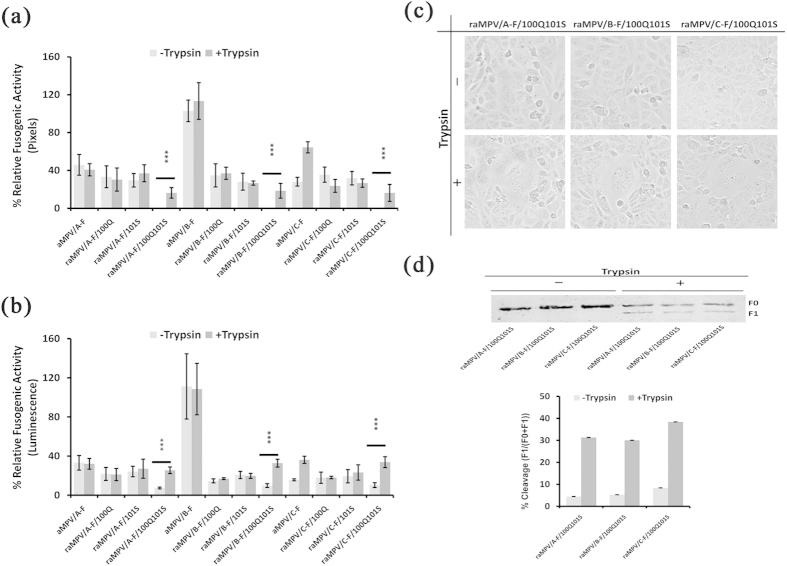
The fusogenic activity of aMPV F proteins in the presence or absence of trypsin. (**a,b**) The fusogenic activity of wild-type and mutant aMPV F proteins in the presence or absence of trypsin was assessed through syncytium formation and reporter gene assays. *P* < 0.05 and *P* < 0.005 are represented as **and ***, respectively. (**c**) Analysis of mutant 100Q101S aMPV F proteins in mediating cell-cell fusion in the presence and absence of trypsin (magnification: 100×, 8 cm ×8 cm as shown). (**d**) The cleavage of mutant aMPV F proteins containing 100Q101S was analyzed through biotinylation and western blot analyses. The data represent the ratio of the expression of F1 vs. the expression of F0 and F1.

A previous study indicated that hMPV, harboring the putative cleavage motif (99-RQSR-102), was incapable of initiating growth without exogenous trypsin; however, the S-to-P substitution (S101P) facilitated virus growth in a trypsin-independent manner, suggesting that the residue at position 101 promotes the cleavage of hMPV F protein and subsequent infection with hMPV^19^. In addition, the results indicated that the F proteins of all aMPV strains mediate membrane fusion in the absence of trypsin, partly because the precursor protein F0 can be cleaved without trypsin treatment (Fig. 1). Sequence alignment analyses (Fig. 2a,b) and previous studies^18^ have shown that the cleavage region of aMPV F protein is located at positions 99 to 102. To further define the residues in the cleavage region of aMPV F protein that might confer the distinct fusogenicity of aMPV subtypes, the F protein sequences of some representative aMPV/A, aMPV/B, aMPV/C, and hMPV strains were aligned. The results of the alignment analysis suggested that the residues at positions 100 or 101 in the putative cleavage region might be determinants for aMPV F protein-mediated cell-cell fusion in the absence of trypsin. Site-directed mutagenesis was therefore performed to replace one or both of these residues in the F proteins of aMPV/A, aMPV/B, and aMPV/C with 100Q or/and 101S, resulting in the plasmids indicated in Fig. 3a. Syncytium formation and reporter gene assays were conducted in the presence or absence of trypsin, as described above. The results consistently showed that single mutations at either position 100 or 101 did not lead to significant differences in the fusogenicity of aMPV F proteins in the presence of trypsin compared with that without trypsin for each aMPV subtype (Fig. 3a–c). This result suggested that single point mutations at either site (100 or 101) did not alter the trypsin-independent phenotype of aMPV F proteins. Interestingly, the substitution of both amino acids at positions 100 and 101 ablated or dramatically decreased the fusogenic activity of all F proteins, resulting in a trypsin-dependent phenotype for all aMPV subtypes (Fig. 3a–c). These results were further confirmed through biotinylation and western blot analyses (Fig. 3d), suggesting that aMPV mutant F proteins containing 100Q101S substitutions can be cleaved only after treatment with trypsin. Together, these findings demonstrated that the two residues at positions 100 and 101 in aMPV F proteins were determinants for aMPV F protein-induced membrane fusion in the absence of trypsin and that the addition of trypsin boosted the fusogenic activity of aMPV F 100Q101S mutants of all examined aMPV subtypes.

### Trypsin promotes the fusogenic activity of aMPV F proteins with 100 K101A mutations

**Figure 4:**
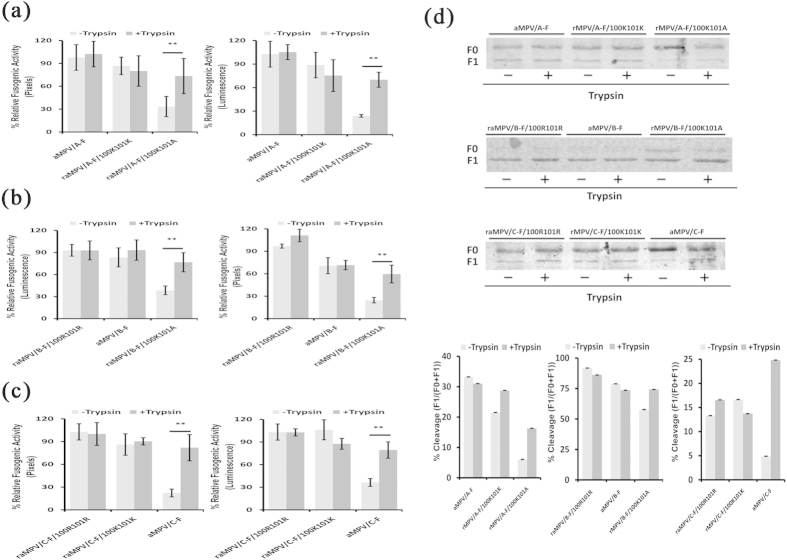
Effect of the residues at positions 100 and 101 on the fusogenic activity of aMPV F proteins. (**a–c**) Analysis of the fusogenic activity of mutant and wild-type aMPV F proteins using syncytium formation and reporter gene assays. *P* < 0.05 and *P* < 0.005 are respectively presented as **and ***. (**d**) Analysis of the expression of mutant and wild-type aMPV F proteins using biotinylation and western blot analyses. The data represent the ratio of the expression of F1 vs. the expression of F0 and F1.

As described above, the results showed that trypsin enhanced the fusogenicity of aMPV/C F protein, but not aMPV/A or aMPV/B F. To determine the mechanism of the observed difference in cell-cell fusion induced through F proteins of different aMPV subtypes, we reciprocally replaced the residues at positions 100 and 101 of the aMPV/A, aMPV/B, and aMPV/C F proteins and examined the effect of trypsin on fusion promotion. As shown in Fig. 4a–c, in the absence of trypsin, the fusogenicity of all mutant and wild-type aMPV F proteins containing 100 K101A was lower than that of F protein harboring 100R101R or 100 K101 K. Interestingly, the fusogenic activity of all mutant and wild-type aMPV F proteins containing the motif 100 K101A, which is characteristic of aMPV/C, was considerably increased with the treatment of trypsin. In addition, the cleavability of all expressed aMPV F proteins was validated through biotinylation and western blotting, as described above. The results indicated that the cleavability of all mutant and wild-type aMPV F proteins possessing the 100 K101A mutation was reduced without trypsin treatment compared with that of the respective proteins under trypsin treatment (Fig. 4d). Thus, trypsin slightly enhanced the cleavability of aMPV F protein containing 100 K101A, which in turn resulted in the enhanced fusogenic activity of mutant F proteins of aMPV/A or aMPV/B to a level comparable to that of the aMPV/C F protein.

### Low pH-induced cell-cell fusion mediated through the aMPV/C F protein

**Figure 5:**
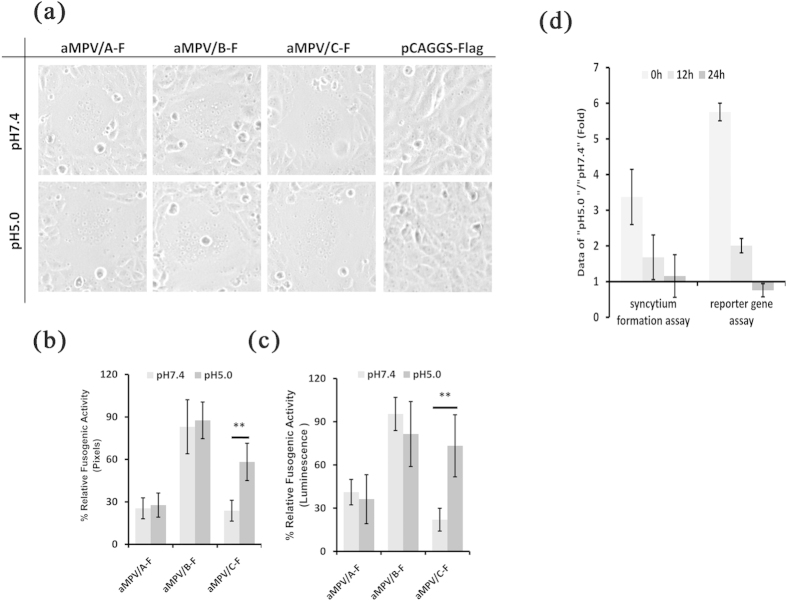
The effect of low pH on membrane fusion induced through aMPV F proteins. (**a**) Syncytium induced through aMPV F proteins at low or neutral pH (magnification: 100×, 8 cm ×8 cm as shown). (**b,c**) The fusogenic activity of aMPV F proteins exposed to low or neutral pH was analyzed through syncytium formation and reporter gene assays. *P* < 0.05 and *P* < 0.005 are respectively presented as **and ***. (**d**) The time interval between transfection and low pH treatment influences the fusogenic activity of aMPV/C F protein. aMPV/C F protein was treated with low pH at 0, 12, or 24 h post-transfection. At 48 h post-transfection, the fusogenicity of aMPV/C F protein was analyzed. The data are presented as the ratio of the aMPV/C F protein at low pH vs. aMPV/C F protein at neutral pH.

Fusion between the viral and endosomal membranes occurs in a low pH environment, whereas fusion with the plasma membrane is typically induced through neutral pH^20^,^38^,^43^,^50^. To examine the fusogenic activity of aMPV F proteins in different pH environments, Vero cells transfected with aMPV/A-F, aMPV/B-F, or aMPV/C-F were treated with PBS at pH 5.0 or pH 7.4. As shown in Fig. 5a, all aMPV F proteins induced syncytium formation at neutral pH after 48 h of transfection (Fig. 5a). In contrast, no syncytium was observed in Vero cells transfected with empty vector after neutral or low pH treatment (Fig. 5a). However, after low pH treatment, the syncytium induced through aMPV/C F protein increased in number (data not shown) and size (Fig. 5a) compared with that after neutral pH treatment. Moreover, there was no significant difference in syncytium number (data not shown) and size (Fig. 5a) between neutral and low pH treatments for aMPV/A and aMPV/B F proteins. Using quantitative assays, we further analyzed the effect of low pH on the fusogenic activity of aMPV F proteins. The results showed that the fusogenicity of aMPV/C F protein was remarkably increased after low pH treatment compared with that of neutral pH treatment, whereas low pH had no influence on the fusogenic activity of aMPV/A and aMPV/B F proteins (Fig. 5b,c). These results showed that, unlike aMPV/A and aMPV/B F proteins, aMPV/C F protein fusogenicity was induced through low pH.

The results that low pH promoted the fusogenic activity of aMPV/C (Colorado strain, accession number: AY590688.1) F protein are inconsistent with the results of Wei Y *et al.*, who demonstrated that the same strain F protein mediated membrane fusion independent of low pH^18^. According to published studies on the time interval between transfection and low pH treatment^20^,^38^,^39^, we hypothesized that different time intervals might lead to differences in fusogenic activity. To verify this hypothesis, we treated aMPV/C F protein with low pH at 0, 12, or 24 h post-transfection. At 48 h post-transfection, we then analyzed the fusogenicity of aMPV/C F protein. Interestingly, the fusogenicity of aMPV/C F protein under low pH treatment was only significantly higher than that at neutral pH treatment at a time interval of less than 12 h. In contrast, no significant difference in the fusogenic activity was observed when the time interval was more than 12 h (Fig. 5d). When the time interval exceeded 24 h, consistent with Wei Y *et al.*^18^, the fusogenic activity of aMPV/C F protein was similar at low and neutral pH (Fig. 5d). These results partially explain the discrepancy between the results obtained in the present study and those of Wei Y *et al.*^18^.

### Residue 294G was the determinant for aMPV/C F protein-mediated low pH-dependent cell-cell fusion

**Figure 6:**
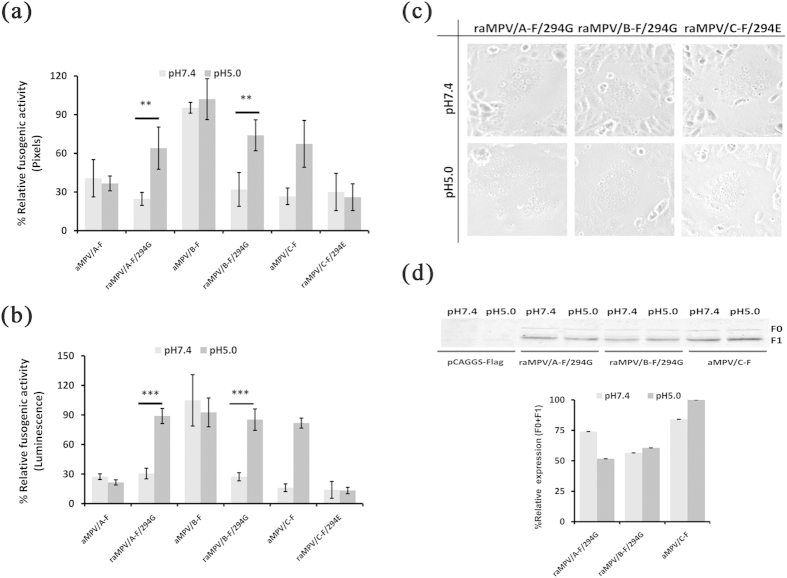
Fusogenic activity of mutant aMPV F proteins exposed to low or neutral pH. (**a,b**) The fusogenic activity of aMPV F proteins exposed to low or neutral pH was measured using syncytium formation and reporter gene assays. *P* < 0.05 and *P* < 0.005 are respectively presented as **and ***. (**c**) Syncytium induced through mutant aMPV F proteins at low or neutral pH treatment (magnification: 100×, 8 cm × 8 cm as shown). (**d**) The expression of wild-type and mutant aMPV F proteins exposed to low pH or neutral pH was analyzed through biotinylation and western blot analyses.

Low pH-induced cell-cell fusion mediated through aMPV F proteins was subtype-dependent (Fig. 5) and strain-dependent for hMPV F proteins^39^. For hMPV, residues 294E and 294G are responsible for wild-type F protein-mediated membrane fusion in low pH-independent and -dependent manner, respectively^39^. Interestingly, the alignment analysis of F protein sequences revealed that the aMPV/C F protein, a low pH-dependent phenotype, possesses residue 294G, whereas aMPV/A and aMPV/B F proteins, both low pH-independent phenotypes, did not possess a glycine (G) at position 294 (Fig. 2a,b). We therefore examined whether substitution of G294 to E was sufficient to change the low pH-dependent phenotype of aMPV/C F protein into a low pH-independent phenotype. Site-directed mutagenesis was performed to change the G residue at 294 in aMPV/C F protein to an E residue, and the fusogenic activity of mutant and wild-type aMPV/C F proteins was compared. The results demonstrated that the G294E substitution changed aMPV/C F protein into a low pH-independent phenotype (Fig. 6a–c), suggesting that 294G mediated the low pH induction of aMPV/C F protein fusogenicity.

To determine whether residue 294G is also responsible for the low pH-dependent phenotype in the aMPV/A and aMPV/B F proteins, we mutated the 294 site of aMPV/A and aMPV/B F proteins into glycine (G) and detected the fusogenic activity of wild-type and mutant F proteins, as described above. As expected, the mutant aMPV/A and aMPV/B F proteins containing 294G, the same amino acid same as observe in aMPV/C F protein, mediated cell-cell fusion in a low pH-dependent manner (Fig. 6a–c). Taken together, these results indicated that residue 294G is a key determinant for mediating cell-cell fusion in a low pH-dependent manner for aMPV F proteins. Furthermore, we performed biotinylation and western blot analyses to exclude the possibility that the low pH environment affects the expression of aMPV F proteins, and the results showed that the expression was similar at neutral and low pH (Fig. 6d).

### 294 site regulated the fusogenic activity of aMPV F proteins

**Figure 7:**
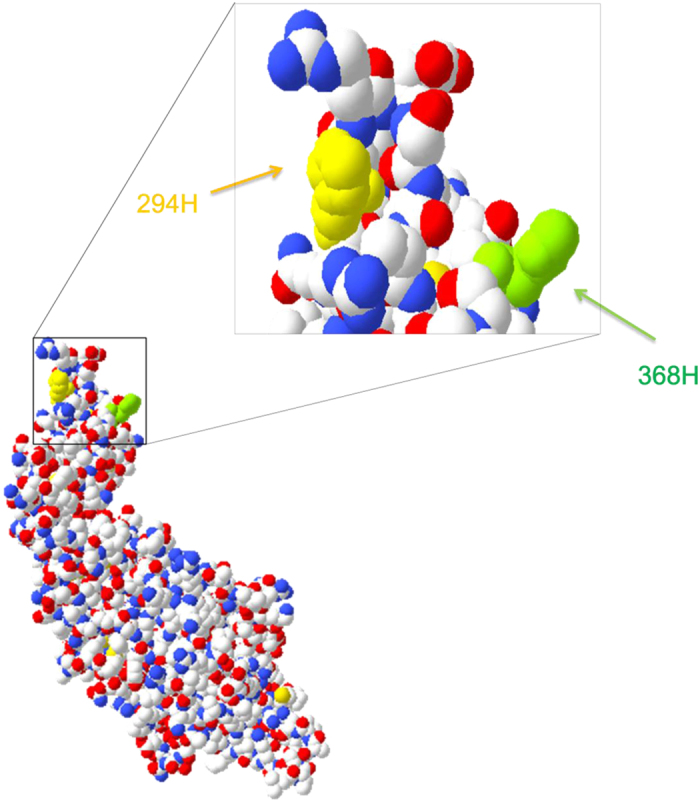
Location of critical residues affecting the fusogenic activity of aMPV F protein at low pH. The model represents the conformation of the aMPV/B F protein, generated using the atomic coordinates of the preactive hMPV F protein structure, consistent with Wen *et al.*^52^, using the SWISS-MODEL server (http://swissmodel.expasy.org/).

**Figure 8:**
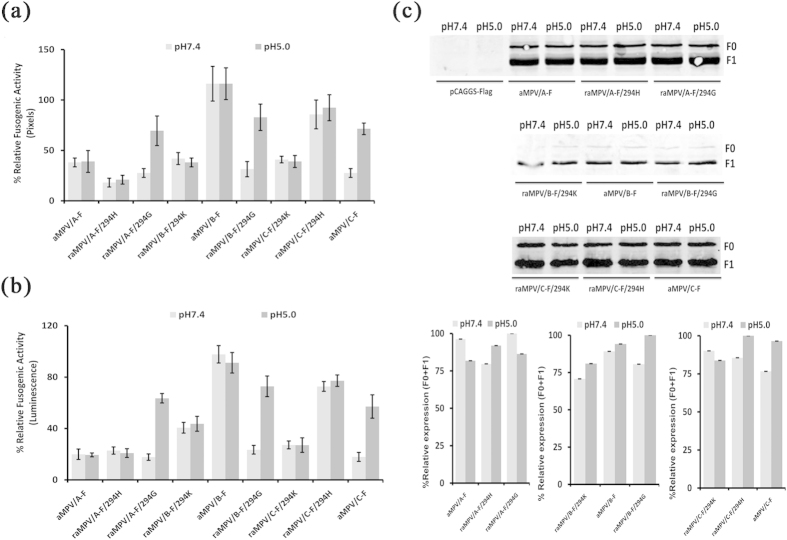
Effect of the residue at position 294 on the fusogenic activity of aMPV F proteins. (**a,b**) The fusogenic activity of wild-type and mutant aMPV F proteins exposed to low or neutral pH was measured using syncytium formation and reporter gene assays. (**c**) The expression of wild-type and mutant aMPV F proteins was analyzed through biotinylation and western blot analyses.

The protonation of histidines and electrostatic interactions are important for the activation of certain F proteins^38^,^39^,^51^. Previous studies have also demonstrated that residue 294G is responsible for the low pH-stimulated promotion of the fusogenic activity of hMPV F proteins through the regulation of protonation at 368H^39^. Because the 294 site of aMPV F protein is located near residue 368H in the three-dimensional model^39^,^52^ (Fig. 7), we predicted that 294 site might also affect the fusogenic activity of aMPV F proteins. To examine this hypothesis, the residues at 294 were reciprocally interchanged among aMPV/A, aMPV/B, and aMPV/C F proteins, and the fusogenic activity of these mutants was compared with that of the wild-type F proteins. The results showed that the fusogenic activity of aMPV/B and aMPV/C F proteins was increased in the 294H derivatives compared with that of the 294 K and 294G derivatives (Fig. 8a,b). However, this result was not observed for aMPV/A F protein (Fig. 8a,b). Taken together, the 294 site was identified as an important factor that affects the fusogenic activity of aMPV F proteins. Furthermore, we performed biotinylation and western blot analyses to exclude the possibility that substitutions at the 294 site alter the expression of aMPV F proteins, and the results showed that the expression was similar among the 294 K, 294H, and 294G derivatives (Fig. 8c).

## Discussion

The entry of aMPV into host cells requires the initial fusion of viral and cell membranes mediated through F protein^18^,^53^. The dissection of the basic requirements for aMPV F protein-induced cell-cell fusion has facilitated studies on aMPV entry; however, the mechanism of aMPV F protein-mediated trypsin- and low pH-induced membrane fusion remains unclear. Here, we revealed that trypsin is not essential for the fusogenic activity of aMPV F proteins and that low pH-induced membrane fusion is subtype dependent. Moreover, we have provided the first evidence that the residues at positions 100, 101 and 294 affect the fusogenic activity of aMPV F proteins regulated through trypsin and low pH treatments, indicating that these residues might influence aMPV virulence and entry.

The cleavage of precursor F protein (F0) is a prerequisite for F protein-mediated membrane fusion, which in turn mediates viral entry and infection^19^. In the present study, we revealed that aMPV/A, aMPV/B, and aMPV/C F proteins induce cell-cell fusion in the absence of trypsin. Site-specific mutagenesis and quantitative assays for fusogenic activity demonstrated that the residues at positions 100 and 101 in the putative cleavage motif were responsible for aMPV F protein-mediated cell-cell fusion without the exogenous addition of trypsin (Fig. 4). Previous studies have shown that for NDV, hPIV2, and Sendai virus *in vivo*, the trypsin-dependent cleavage motif is only recognized via tissue-specific proteases, whereas trypsin-independent cleavage motifs are typically recognized via nonspecific ubiquitous proteases, thus increasing host range and virulence^19^. In contrast with the trypsin-dependent cleavage motif, whether the trypsin-independent cleavage motif promotes aMPV infectivity and virulence in turkeys and chickens is unclear, and further studies are warranted.

In general, paramyxovirus F proteins containing multibasic residues at the cleavage site are easily cleaved via intracellular proteases, whereas F proteins containing monobasic residues require exogenous trypsin for cleavage^19^. The putative cleavage motif of aMPV/A and aMPV/B F proteins comprises basic residues, while the cleavage site of aMPV/C F harbors a neutral alanine (A). Therefore, trypsin increased the fusogenic activity of aMPV/C F_100K101A_ protein but had no effect on aMPV/A F_100R101R_ and aMPV/B F_100K101K_ proteins (Fig. 1).

Studies investigating the mechanism of how low pH activates cell-cell fusion would greatly enhance the biophysical understanding of paramyxovirus-induced membrane fusion^51^. Here, we demonstrated that low pH stimulated the fusogenic activity of aMPV/C F protein in contrast to that observed with aMPV/A and aMPV/B F proteins. The reciprocal interchange between residues at position 294 in aMPV/A, aMPV/B, and aMPV/C F proteins demonstrated that residue 294G was responsible for the increased the fusogenic activity of aMPV F proteins at low pH (Fig. 6 and 8). Paramyxovirus uses a neutral pH for plasma membrane entry routes^38^,^43^, whereas the results obtained in the present study suggest that residue 294G might alter the entry pathway of aMPV to that of a low pH endocytic route. Further study is needed to define the entry pathways for aMPV/A, aMPV/B, and aMPV/C.

There was no clear explanation for why residue 294G determines the low pH-dependent phenotype of aMPV and hMPV F proteins. A previous study suggested that residue 294G induces the protonation of 368H in F proteins induced at low pH^39^. Additionally, aMPV/B and aMPV/C F proteins carrying residue 294H are more fusogenic than those carrying 294G and 294 K, and the same applies to hMPV (Canada 97–83 strain, accession number: AY297749.1) F proteins (data not shown) but not aMPV/A F protein (Fig. 8). The mechanism underlying the effect of position 294 on the fusogenicity of aMPV and hMPV F proteins requires further study.

Sequence analyses have shown that aMPV/C is more closely related to hMPV than to aMPV/A and aMPV/B^54^,^55^. Recently, aMPV/C and hMPV have been subclassified into the same type based on phylogenetic analysis, while aMPV/A and aMPV/B belong to another type^5^. In addition, aMPV/C has limited cross-reactivity with aMPV/A and aMPV/B in virus neutralization assays^56^. Furthermore, aMPV/C has closer antigenic and genetic relatedness to hMPV than to aMPV/A and aMPV/B^57^,^58^. Interestingly, Wei *et al.* demonstrated that the behavior of aMPV/C in mice is similar to that of hMPV, suggesting that aMPV/C might emerge as a zoonotic pathogen^56^. We also showed that the mechanism of trypsin- or low pH-induced membrane fusion mediated through aMPV/C differs from that of aMPV/A and aMPV/B and is more similar to some hMPV strains. Because aMPV F protein-mediated membrane fusion induces viral infectivity, the distinctive fusion mechanism of aMPV/C F protein might produce different patterns of pathogenicity in aMPV/C relative to aMPV/A and aMPV/B but confer similarity to hMPV.

In summary, we demonstrated that positions 100 and 101 primarily determine aMPV/A, aMPV/B, and aMPV/C F protein-mediated cell-cell fusion in the absence of trypsin and that residues 100 K101A determine the trypsin-induced fusogenic activity of aMPV/C F protein. In addition, we also demonstrated that residue 294G influences aMPV/C F protein-induced cell-cell fusion in a low pH-dependent manner. Further research on this aspect will shed light on the mechanism of aMPV F protein-mediated membrane fusion and substantially contribute to a better understanding the pathogenesis of aMPV.

## Methods

### Cells and Plasmids

Vero cells and BHK-21 cells were grown in Dulbecco’s modified Eagle’s medium (DMEM; Invitrogen/Life Technologies) supplemented with 10% fetal bovine serum (FBS, Gibco, Invitrogen/Life Technologies) and 1% penicillin and streptomycin (summus, Beijing, China). The F gene of aMPV strains LAH A strain (aMPV/A), VCO3/60616 strain (aMPV/B), and Colorado strain (aMPV/C) was amplified by RT-PCR and then subcloned into the pCAGGS mammalian expression vector by using the *Kpn* Ι and *Xho* Ι restriction sites, resulting in plasmids aMPV/A-F, aMPV/B-F, and aMPV/C-F. Flag tag fusing with F gene was introduced into each of these plasmids above for identification. All aMPV F gene mutants were created with In-Fusion^®^ HD Cloning Kit (Clontech, Mountain View, CA, USA) using aMPV/A-F, aMPV/B-F, and aMPV/C-F as template. All aMPV wild-type and mutant F genes used in this study were verified by automated DNA sequencing.

### Syncytium formation assay

Monolayers of Vero cells in 6-well plates were transfected with 2 μg of each plasmid using Lipofectamine 2000 (Invitrogen, Carlsbad, CA, USA) according to the manufacturer’s instructions. The transfected cells were incubated with or without 0.2 μg/mL TPCK (L-1-tosylamide-2-phenylethyl chloromethyl ketone)-trypsin (Sigma) for up to 24 h. After transfection, the cells were exposed to phosphate-buffered saline (PBS) at pH 5.0 (low pH) or pH 7.4 (neutral pH) for 4 min as previously described^20^,^39^. This pH pulse was repeated three times at 2-h intervals. At 48 h post-transfection, the cells were observed and photographed. The areas involved in syncytium formation were selected from representative photographs of the experimental samples. These areas were subsequently compared with the total analyzed area using the Magnetic Lasso Tool and histogram function of Adobe Photoshop software, and the percentage of cells forming syncytia was calculated to evaluate the fusogenic activity as previously described^44^,^45^.

### Reporter gene assay

The reporter gene assay was performed to evaluate the fusogenic activity of aMPV F proteins according to previous reports^8^,^39^. Lipofectamine 2000 was used to transfect Vero cells in 6-cm dishes with 2 μg of wild-type or mutant aMPV-F and 1 μg T7 control plasmid (Promega, Madison, WI) containing luciferase cDNA under the control of the T7 promoter. The transfected Vero cells were incubated with or without 0.2 μg/mL of trypsin for up to 24 h. The transfected Vero cells and BHK-21 cells infected with MVA-T7 at a multiplicity of infection (MOI) of 1 were treated with PBS (pH 5.0 or pH 7.4) after transfection. The next day, the Vero cells were lifted, resuspended and overlaid onto infected BHK-21 cells. At 48 h post-transfection, the cell lysates were analyzed for luciferase activity using a luciferase assay system (Promega) according to the manufacturer’s instructions. The luciferase activity was measured on an EnVision Multilabel Reader (PerkinElmer, Waltham, MA).

### Biotinylation and Western blotting

Expression of aMPV F proteins on the cell surface was measured by biotinylation and western blotting, as described previously^38^,^46^. The transfected Vero cells were biotinylated with membrane-impermeable EZ-Link Sulfo-NHS-SS-Biotin (Thermo Scientific, Rockford, IL). The cells were lysed in Cell Lysis Buffer for Western and IP (Beyotime, Beijing, China) and centrifuged to collect the supernatants, which were immunoprecipitated with streptavidin beads (Thermo Scientific). The expressed proteins were separated by SDS-PAGE and transferred to a nitrocellulose membrane (Hybond-C Super; GE Healthcare, Piscataway, NJ). The nonspecific antibody-binding sites were blocked with 5% skim milk in PBS containing 1% Tween-20. Monoclonal anti-flag antibody (Sigma) diluted (1:2,000 dilution) in PBS containing 1% Tween-20 was used to react with target protein. The second antibody, IRDye 800CW goat anti-mouse IgG (H + L) (LiCor BioSciences, Bad Homburg, Germany) (1:5,000 dilution), was added, followed by incubation in a dark place at room temperature for 1 h. The nitrocellulose membranes were exposed to LI-COR ODYSSEY (LiCor BioSciences) for visualization of aMPV F proteins. The cleavage extent and expression level of F proteins were evaluated by software Gelpro32.

### Statistical analysis

Statistical analyses were performed using SPSS 13.0 software. The data represent the means (±standard error) of at least three experiments. A *P* value (one-way analysis of variance, two-tail paired t-test) of <0.05 was considered significant.

## Additional Information

**How to cite this article**: Yun, B. *et al.* Trypsin- and low pH-mediated fusogenicity of avian metapneu-movirus fusion proteins is determined by residues at positions 100, 101 and 294. *Sci. Rep.* **5**, 15584; doi: 10.1038/srep15584 (2015).
